# Revisiting the need for virtue in medical practice: a reflection upon the teaching of Edmund Pellegrino

**DOI:** 10.1186/s13010-018-0057-0

**Published:** 2018-04-10

**Authors:** Luchuo Engelbert Bain

**Affiliations:** 1 Centre for Population Studies and Health Promotion, CPSHP, Yaounde, Cameroon; 2 Athena Institute for Research on Innovation and Communication in Health and Life Sciences, Vrije Universiteit Amsterdam, Amsterdam, The Netherlands

**Keywords:** Edmund Pellegrino, Ethics, Morality, Virtue, Art of medicine

## Abstract

Edmund Pellegrino considered medicine as a skill, art, and perhaps most importantly, a moral enterprise. In this essay, I attempt to exemplify how the legacy and contributions of Edmund Pellegrino, as a teacher and a physician, could allow for a renaissance of medical practice in which physicians engage intellectual and moral virtue to both effect sound care, and do so in a humanitarian way, rather than in simple accordance with a business model of medicine. The virtues are viewed in a renewed light as being key characteristics of physicians, and important to patient centered care.

“No matter to what depths a society may fall, virtuous persons will always be beacons that light the way back to moral sensitivity; virtuous physicians are the beacons that show the way back to moral credibility for the whole profession”

*Pellegrino ED. The Virtuous Physician, and the Ethics of Medicine. In: Virtue and Medicine Springer, Dordrecht; 1985. p. 237–55.*

## Background

A 23 year old frail and kind hearted lady living with HIV - AIDS on combined anti retroviral therapy (cART) was seen in my office at the outpatient department. After greetings and a clinical discussion for over 25 min on the challenges she faces with her condition, my patient tells me she has nothing to add or ask, at least for now. I then insist and ask if she had some other things she might want to discuss, or maybe, that I failed to address a concern she would have wished to talk about. She nodded her head and burst into tears. Feeling challenged and guilty, thinking I had hurt her unknowingly with this seemingly innocent question in good faith, I supported her and finally asked if there is something wrong, and assured her that if I hurt or offended her, I was sorry. She wiped her tears and voiced what was perhaps one of the most touching sentences I ever heard in my humble career as a primary care physician: “At last, someone will finally allow me talk, and be listened to”.I could not help regretting how many patients, in my innocence, were disappointed by my trying to use more of the scientific components of being a physician, and failing to recognize that medicine in itself was, is and remains an art at its very core. Moreover, I find it saddening to perceive what may be a growing divide in the patient – physician relationship.

### Edmund Pellegrino, physician and teacher

The late Edmund Pellegrino, physician, scholar, and renowned bioethicist who recognized and espoused the humanities as integral to medicine, is considered to be a leading figure in advocating the virtues in and for medical practice [1, 2]. Pellegrino believed that one undergoes an existential change when one becomes a patient. For Pellegrino, the clinical encounter was seen as having been increasingly reduced to pre-defined formulae to obtain exacting results. I agree, and claim that the absence of time and the centrality of technology are key drivers in medical encounters, and have eroded much of the humanitarian aspect of clinical medicine. The empathic relationship that physicians and patients once enjoyed is fast fading [3–5]. How disappointing to recognize that the ideals of the art of medicine, as a moral practice, are being increasingly transformed into a technologically-focused, business-oriented interaction.

While technology is important, patients are often dissatisfied with use and interpretation of technologically-based assessments and interventions. The philosopher Emmanuel Levinas exemplified the medical encounter as one where the physician sees the “other” as the very reason of her/his purpose: Simply put, without the patient, the physician is little more than a biomedical scientist. This reliance upon “the other” creates a practice paradigm where the suffering of the patient provides the moral impulse for the physician’s goals and aims of care, to be articulated in those ways in which she/he would have wanted to be treated [6]. This is congruent with Kant’s categorical imperative, wherein our actions toward others should be congruent with what we would expect of them toward us if in a similar circumstance. An empathic exchange or relationship with the patient establishes a trusting ground from which the physician is able to engage her/his acts of inquiry, examination and treatment [6]. In many ways, this empathy- taken together with technical skill- builds patient trust. Such trust is important both to fostering patients’ comfort in divulging information relevant to their history and concerns, and in supporting their compliance with recommended treatments.

Physician and ethicist Joseph Fins has described the life of Edmund Pellegrino as one full of virtue, commitment, consistently living what he preached, and always having the patient at the center of his deeds as a physician and ethicist [7, 8]. However, the widening acceptance of a business-oriented utilitarian ethics has in some ways lessened the Hippocratic character of medicine as a public good [9]. Physician-philosopher Julian Savulescu assumes a utilitarian view of doctors as simply persons, acting as professionals who must respond to patients’ needs, values and demands [10]. Without doubt, patients’ needs and values are important, but not in abject disregard for the role of physician conscience in objecting to execute patients demands that trump physician autonomy in decisions and actions that incur ethical dissonance [10]. Savulescu regards the physician as a public servant, who should act in the interest of the public (and the patient as a constituent of that public) [10]. Though this move empowers to some extent patient implication in decision making, it might also be seen as diminishing the flexibility to the physician to engage shared decision-making within the context of the healing relationship with her/his patient, and in some ways might misconstrue the most literal construct of patient autonomy – not as a positive right of choice, but as the (negative) right of refusal.

This is important because economic factors are playing an ever increasing role in health care provision, delivery and access. The medical systems of today tend to function in accordance with and response to a market system. Of course, there cannot or should not be disregard for the importance of economics in medical decision-making and health policy. But such regard should not disavow the humanitarian goals of medicine as focusing upon of the patient, which undergirds medicine. In what follows, I will argue that physicians – entailing a fortified virtue ethic - could help to safeguard the interests of the patient in the healing relationship, enhance shared decision making and improve the trust patients have for their doctors.

### Power in listening and empathy

Surely, physicians generally have time constraints, and their professional obligations are divided between clinical ministrations, data entry into computer systems, ward rounds, teaching and research. But I argue that these latter tasks serve, and are subordinate to the former, which is caring for the patient. The most used definition of health as a state of complete physical, social and psychological well-being, and not a mere absence of disease, has become more diffused through a directed focus upon physical components. As a primary care physician, it has become clearer to me that a good number of patients visit doctors just to be listened to. The art of medicine is needed to be able to recognize and act on the social and psychological dimensions of patients’ definition of what constitutes the good(s) of their lives, goals and choices. Contrary to popular opinion, many patients trust physicians, and are ready to disclose sensitive issues, which could even be unknown to their immediate family. A physician lacking the art of active and empathic listening and emotional sensitivity might not win the trust of the patient on such occasions. Pellegrino insisted upon the moral nature of medicine and of being a physician. These obligations distinguish medical care from mere business dealings. Pellegrino identified the commodification of medicine as key factor in the moral erosion of medical practice [7, 8, 11]. The uniqueness of medicine is based in large extent upon the human relationship of physician – as healer, and patient – as one who requires such healing. Active listening is important to this relationship, yet may be fading from the priorities of contemporary medicine. Yet, such listening serves a core process through which to express concerns, dispel fears and create a trusting relationship between the patient and the physician.

I posit that we must remind ourselves and future physicians of Pellegrino’s teaching that medicine is a moral practice. Acknowledging the achievements of advancing technologies need not dictate acceptance of changing medicine from an art and a moral practice, into a mechanical, automated system. Evidence and technological capability are important to the conduct of medicine, but statistics and the use of ever-newer tools can be “sterile” without the pathos of the physician to communicate such information and aptly utilize new technologies in ways that convey sensitive care. Given the increasingly technological trend in medicine, it might be time to question if there is something lacking in the training of physicians when it comes to the human dimensions of the clinical encounter. Do we need another Flexner, perhaps, to tell us that our medicine is taking an unacceptably negative turn, and to revise curricula toward a corrective path?

Alas, I am, like all persons at some point in their lives, also a patient, and I often feel frustrated by my treating physicians’ attendance to a computer, rather than listening, examining or talking to me. This in no way reduces the importance of data within the practice of the art. Surely, data support diagnoses and directions for care. Furthermore, data are crucial to insurance programs that support such care. But data should be used to inform and guide medical decisions and care that are focused upon the good of the patient. It is easy to point to the ways that certain insurance schemes exert direct impact on quantity and quality of care that physicians can provide – and which patients can access.

But if there has been the erosion of practice – and the Hippocratic maxim to honor that which has been passed down as the tradition of medicine – and if we accept that such erosion is regarded as deleterious to the nature, scope and value of medicine and human health, then where rests the responsibility to change this negative trend? I argue that if we view medicine as a moral practice, and evidence and acknowledge its moral erosion, then it is (our) incumbent moral responsibility to effect repair by reviving those traditions and practices of value, and training a new generation of virtuous physicians.

### A clinical encounter, and not a “consultation”: Medicine as a moral enterprise

The recognition of the patient as the essential focus of the physician’s presence, and as the prompt for her/his sensitivity and response to the patient in front of her/him, must continue to be primary to the conduct of medicine in practice. Patients are unique human beings, and while clinical data and research findings are important, caution and pathos must prevail when using information and technology at the bedside. I am inclined to believe that a sound employ of intellectual and moral virtues could reverse the trend away from such respected tenets, and preserve medicine as a moral enterprise. Pellegrino reminded us of the virtues of a good physician: fidelity, trust, benevolence, intellectual honesty, courage, compassion, truthfulness – and practical wisdom [2, 11].

Like Aristotle, Pellegrino firmly believed that virtues can be taught [12, 13]. He recognized however, that this is not an easy task. As a moral practice however, Pellegrino maintained that teaching virtue to physicians remains a mandate in order to safeguard the *telos* of the art. Pro Pellegrino, Shelton proposed that teaching virtue should be an institutional mission of medical education [14]. Contrasting arguments pose that individualism could be a potential barrier to developing curricula to teach virtue. However, in reflecting upon what the “good doctor” should be, Shelton describes a professional who exercises respectful interactions with the patient, is an empathic listener, and appreciates patients’ narratives [14]. He acknowledges individualism, and in this light encourages medical teachers to stimulate flexibility and creativity in and among their trainees. This builds upon Pellegrino’s telic view of medicine as framed by the recognition of circumstance in establishing “what is the right and good healing act for this patient?” [12, 15]. Recognition of the importance of the circumstance can be seen as an opportunity for the physician’s (and physician trainee’s) creativity and flexibility to further recognize and appreciate the fact that each patient is unique, within an individual life world and lived experience., which needs to be regarded in and when making medical decisions.

Pellegrino and Thomasma describe four levels of the good: the biomedical good, the patient’s perception of his or her good, the good of the patient as a human being and the spiritual good of the patient [12, 13, 15]. The (bio)medical good is a single component of the complex dynamic of the healing relationship. Properly acting upon the other levels of good requires building of a trusting relationship between the patient and the physician. Excluding the views, understanding, appreciation and feelings of the patient in deciding upon “the good of the patient” might constitute a dangerous aspect of paternalism that dilutes the trust of the healing relationship.

The erosion of the moral fabric of medicine could partly explain the increasing number of disappointed patients. If a goal of medicine is the restoration of health, and health represents some construct of the functional whole of the person, then arguably there must be an engagement with the social dimensions of patients’ lives that are influential to their health [4, 16]. In her book, *How Doctors Think*, Montgomery gives special attention to the role of skilled listening in remaking medical practice as an art [4]. Masel et al., report that palliative care patients expect and identify that a “good” physician should be an attentive listener, should be honest, experienced, gentle, and humane [17]. Surely, Pellegrino’s definition of medicine as a moral enterprise centered upon the healing relationship could obtain most, if not all of these patient expectations [11]. But this fosters the question of whether we uniformly screen for, and/or teach these qualities in medical schools. These qualities, or to be more precise – virtues - need to be reactivated and inculcated in the physicians of tomorrow [3].

The scientific knowledge of the physician becomes a uniquely meaningful contribution to the patient’s care and healing through active and empathic listening, respectful clinical examination, and discussion of treatment options with relevance to the patient’s life and choices. Physicians must therefore appreciate the physician – patient encounter, not merely as consultation, but as a discussion to learn from the experience of the vulnerable patient, and respond appropriately with use of medical science, with the active participation of the patient in the decision making process. Pellegrino generally avoided the use of the term “medical consultation”, preferring instead the “clinical encounter”. And an encounter it should be. Central to Pellegrino’s ethic of the culture of healing is the physician’s ability to appropriately distance one’s self so as to remain enjoined but yet sufficiently separate, so as to prescind and enable a view of the threats to the existential priorities of the patient, and to act both with and for the patient, to address them [18].

In his regular use of the “clinical encounter” Pellegrino emphasized the interactive roles of the patient and physician in the medical decision making process, as well as the inseparability of medicine and philosophy. This encountering enables a forum in which the patient is empowered to define the good of treatment with her/his physician. It is only from such a grounding that the patient can feel comfortable to open her/his life world for the physician to apprehend and use as a guide in those decisions that affect the patient’s healing and health within the framework of their particular values and goals. Evidence based clinical decision making should incorporate all of these aspects, and certainly cultivation of both intellectual as well as moral virtues are important to the sound use of evidence in patient-centered care. Leffel et al. have also advocated the importance of cultivating a culture of virtue in medical training and in the application of clinical skills [19]. For Pellegrino, technical skill, an intellectual virtue, needs to be embraced within an appreciation for and sustainment of moral virtue in medical practice. This is supported by the work of Arthur and colleagues that demonstrated that physicians’ characterization of an “ideal doctor” most often portrays enactment of intellectual and moral virtues in practice [20]. Similar findings have been reported by Kotzee et al. [21]. According to Gardiner [22], “…*a good person who behaves well must develop virtues, which, through habitual use, become part of that person’s character”.*

## Conclusion

Pellegrino is remembered as one of the major advocates of medical practice as a moral enterprise, for virtuous practitioners, with the patients’ good being at the center of care. In this essay, I have tried to exemplify how a virtue based approach could reignite the trust that patients have for their physicians. According to Pellegrino’s view, the healing relationship stands to substantively gain with the active implication of the patient in the definition of his/her good. Medicine as a virtuous art, fosters empathic listening, emotionally sensitivity and the recognition of the uniqueness of each patient, in properly demarcating the patients’ biomedical, psychological and existential needs.

## Abbreviations

AIDS: Acquired Immune Deficiency Syndrome
cART: Combined Anti Retroviral Therapy
HIV: Human Immunodeficiency Virus
